# “Missing” acute coronary syndrome hospitalizations during the COVID‐19 era in Greece: Medical care avoidance combined with a true reduction in incidence?

**DOI:** 10.1002/clc.23424

**Published:** 2020-07-21

**Authors:** Michail I. Papafaklis, Christos S. Katsouras, Grigorios Tsigkas, Konstantinos Toutouzas, Periklis Davlouros, George N. Hahalis, Maria S. Kousta, Ioannis G. Styliadis, Konstantinos Triantafyllou, Loukas Pappas, Fotini Tsiourantani, Efthymia Varytimiadi, Zacharias‐Alexandros Anyfantakis, Nikolaos Iakovis, Paraskevi Grammata, Haralambos Karvounis, Antonios Ziakas, George Sianos, Dimitrios Tziakas, Evgenia Pappa, Anna Dagre, Sotirios Patsilinakos, Athanasios Trikas, Thomais Lamprou, Ioannis Mamarelis, Georgios Katsimagklis, Dimitri Karmpaliotis, Katerina Naka, Lampros K. Michalis

**Affiliations:** ^1^ 2nd Department of Cardiology University Hospital of Ioannina Ioannina Greece; ^2^ Department of Cardiology Patras University Hospital Patras Greece; ^3^ 1st Department of Cardiology “Hippokration” University Hospital Athens Greece; ^4^ Cardiology Department General Hospital “G. Gennimatas” Athens Greece; ^5^ 2nd Department of Cardiology “Papageorgiou” General Hospital Thessaloniki Greece; ^6^ 1st Department of Cardiology “Evaggelismos” General Hospital Athens Greece; ^7^ 2nd Department of Cardiology “Evaggelismos” General Hospital Athens Greece; ^8^ 2nd Department of Cardiology Hellenic Red Cross Hospital Athens Greece; ^9^ Department of Cardiology “Attikon” University Hospital Athens Greece; ^10^ Department of Cardiology University Hospital of Larissa Larissa Greece; ^11^ Department of Cardiology “Sismanogleio” General Hospital Athens Greece; ^12^ Department of Cardiology “AHEPA” University Hospital Thessaloniki Greece; ^13^ Department of Cardiology University Hospital of Alexandroupolis Thrace Greece; ^14^ Department of Cardiology General Hospital “G. Hatzikosta” Ioannina Greece; ^15^ Department of Cardiology “Thriasion” General Hospital of Elefsina Attiki Greece; ^16^ Department of Cardiology “Konstandopoulio” General Hospital Athens Greece; ^17^ Department of Cardiology “Elpis” General Hospital Athens Greece; ^18^ 2nd Department of Cardiology General Hospital of Nikea‐Piraeus “Agios Panteleimon” Piraeus Greece; ^19^ Department of Cardiology 401 Army General Hospital Athens Greece; ^20^ Department of Cardiology Athens Naval Hospital Athens Greece; ^21^ Department of Cardiology New York Presbyterian Hospital/Columbia University Irving Medical Center New York New York USA

**Keywords:** acute cardiac care, acute coronary syndrome, COVID‐19, myocardial infarction, public health

## Abstract

**Background:**

Reports from countries severely hit by the COVID‐19 pandemic suggest a decline in acute coronary syndrome (ACS)‐related hospitalizations. The generalizability of this observation on ACS admissions and possible related causes in countries with low COVID‐19 incidence are not known.

**Hypothesis:**

ACS admissions were reduced in a country spared by COVID‐19.

**Methods:**

We conducted a nationwide study on the incidence rates of ACS‐related admissions during a 6‐week period of the COVID‐19 outbreak and the corresponding control period in 2019 in Greece, a country with strict social measures, low COVID‐19 incidence, and no excess in mortality.

**Results:**

ACS admissions in the COVID‐19 (n = 771) compared with the control (n = 1077) period were reduced overall (incidence rate ratio [IRR]: 0.72, *P* < .001) and for each ACS type (ST‐segment elevation myocardial infarction [STEMI]: IRR: 0.76, *P* = .001; non‐STEMI: IRR: 0.74, *P* < .001; and unstable angina [UA]: IRR: 0.63, *P* = .002). The decrease in STEMI admissions was stable throughout the COVID‐19 period (temporal correlation; R^2^ = 0.11, *P* = .53), whereas there was a gradual decline in non‐STEMI/UA admissions (R^2^ = 0.75, *P* = .026) following the progressively stricter social measures. During the COVID‐19 period, patients admitted with ACS presented more frequently with left ventricular systolic impairment (22.2 vs 15.5% control period; *P* < .001).

**Conclusions:**

We observed a reduction in ACS hospitalizations during the COVID‐19 outbreak in a country with strict social measures, low community transmission, and no excess in mortality. Medical care avoidance behavior is an important factor for these observations, while a true reduction of the ACS incidence due to self‐isolation/quarantining may have also played a role.

## 1 INTRODUCTION

The new coronavirus (SARS‐CoV‐2), which causes the coronavirus disease (COVID‐19) is highly infectious and is responsible for the current pandemic.^1^ Regardless of high or low SARS‐CoV‐2 penetrance among different countries, recommendations on social distancing/self‐confinement and drastic measures restricting the freedom of movement led to an unexpected social experiment for billions of people worldwide. At the same time that the global community was focusing on controlling the spread of COVID‐19 with most countries rapidly redesigning their health services and enforcing unprecedented measures, worries arose about cardiac collateral damage.^2^ Recent reports from countries highly impacted by the pandemic suggest a decline in acute coronary syndrome (ACS)‐related hospitalization rates and primary percutaneous coronary intervention (PCI) activations,^3^, ^4^, ^5^ while epidemiological findings from countries with either high or low COVID‐19 incidence demonstrate variable population mortality rates, which do not always show a substantial increase compared to previous years.^6^ Patients' avoidance of seeking medical care and more conservative ACS management by healthcare systems have been suggested as possible explanations for these observations. Additionally, an unexpected true reduction in the incidence of ACS due to the lack of environmental triggers as a result of the recommendations on social distancing, self‐confinement, and drastic measures restricting the freedom of movement cannot be excluded and needs investigation.

We studied the rate of ACS admissions, treatment strategy, and outcomes during the COVID‐19 outbreak compared to the corresponding period during the previous year in Greece, a country with low penetration of SARS‐CoV‐2 and no excess in overall mortality. Additionally, we assessed the impact of strict social measures on the observed ACS admission rate, and attempted to identify the presence of any cardiac collateral damage.

## 2 METHODS

### 2.1 Design and patient population

We conducted a multicenter, observational, nationwide study on patients admitted with ACS (ST‐segment elevation MI [STEMI], non‐STEMI [NSTEMI], and unstable angina [UA]) to Greek public hospitals with PCI capability, including a primary PCI service. All consecutive patients admitted with a confirmed diagnosis of ACS at discharge were included in this study. Data were retrospectively collected for a 6‐week period from March 2 to April 12 during the COVID‐19 outbreak in 2020 and the corresponding 6‐week control period in 2019. The study complies with the Declaration of Helsinki and was approved by the local ethics committee at each hospital. Written approved consent was waived on the basis of the retrospective use of anonymized patient data.

### 2.2 Study and control periods

The first confirmed COVID‐19 case in Greece was identified on 26 February 2020 and by 2 March 2020, there were only seven confirmed cases. Therefore, we identified 2 March 2020 as the beginning of the COVID‐19 period, and we studied the first 6 weeks of this period until 12 April 2020. During the 6‐week period of the study, the Greek government authorities progressively imposed several measures of social distancing which also contributed to an increase in public awareness: (a) 10 March 2020: lockdown of all schools and universities and recommendation of self‐confinement behavior; (b) 16 March 2020:80% of all business activities were locked down; and, ultimately, (c) 23 March 2020: drastic measures restricting the freedom of movement which continued until 3 May 2020. Until 13 April 2020 (ie, the end of our study period), there was a total of 2145 confirmed COVID‐19 cases and 99 deaths in Greece.^7^

To perform comparative analyses between the COVID‐19 study period and a period without exposure to COVID‐19, the corresponding period during the previous year (2 March 2019 to 12 April 2019) was used as control. Also, the study period was dichotomized to the time before (from 2 March 2020 to 22 March 2020; that is, the first 3 weeks of the study period) and after (from 23 March 2020 to 12 April 2020; that is, the last 3 weeks of the study period) the complete national lockdown, and comparative analyses were also performed for these two time periods during the COVID‐19 outbreak.

### 2.3 Statistical analyses

Categorical variables are presented as counts and percentages, and odds ratios (OR) with 95% confidence intervals (CI), and were compared using the chi‐square or Fisher's exact test as appropriate. Continuous variables included in the analysis did not have a normal distribution and are summarized as median and interquartile range (IQR); comparisons were made using the Mann‐Whitney test. Crude incidence rates (IR) for ACS admissions were calculated by dividing the number of cumulative events by the number of days for each time period. Incidence rate ratios (IRR), comparing the time periods (COVID‐19 vs control period, and period after vs before national lockdown), were calculated using the Poisson regression to model the number of ACS admissions per day. Regression analysis (linear and logarithmic) was used for testing a temporal trend of the relative change (incidence rate ratio) in ACS admissions during the 6‐week period. Linear regression analysis was also performed on the cumulative number of ACS admissions over the 42‐day time period separately for the study and control periods; analysis of covariance was used to compare the slopes of the two regression lines corresponding to the two periods. An alpha level of 0.05 was used to determine statistical significance unless otherwise stated for subgroup analyses; all statistical tests were 2‐tailed. All analyses were done with Stata 10.0 (StataCorp LP, College Station, Texas) and SPSS 17.0 (SPSS Inc., Chicago, Illinois).

## 3 RESULTS

A total of 1848 ACS patients were included in the study (771 during the study period and 1077 during the control period). Detailed descriptive data of the population are presented in Table 1 and Appendix.

**TABLE 1:** Demographics, clinical characteristics, presentation, and angiographic data of patients admitted with ACS in the COVID‐19 and control period

Characteristic/parameter^a^	COVID‐19 (n = 771)		Control (n = 1077)		*P*‐value
Age (years, median [IQR])	64.3 (56‐74)		65 (56‐74)		.30
Female gender	161	(20.9%)	255	(23.8%)	.14
Diabetes	241	(31.4%)	280	(27.5%)	.070
Hypertension	457	(59.4%)	611	(59.8%)	.88
Hypercholesterolemia	423	(55.1%)	573	(56.1%)	.66
Smoking	373	(48.5%)	474	(46.4%)	.37
Family history CAD	133	(17.3%)	154	(14.9%)	.160
Chronic kidney disease	60	(7.8%)	58	(5.6%)	.058
Peripheral arterial disease	43	(5.6%)	57	(5.5%)	.91
Previous CVA	33	(4.3%)	32	(3.1%)	.17
Previous myocardial infarction	163	(21.2%)	199	(18.8%)	.21
Previous PCI	142	(18.5%)	192	(18.1%)	.83
Previous CABG	47	(6.1%)	72	(6.8%)	.57
Admission by interhospital transfer	262	(34%)	352	(32.8%)	.60
ACS presentation					.36
STEMI	247	(32%)	327	(30.4%)	
NSTEMI	352	(45.7%)	479	(44.5%)	
Unstable angina	172	(22.3%)	271	(25.2%)	
MINOCA^b^	53	(7.1%)	76	(7.3%)	.89
Cardiogenic shock	47	(6.1%)	56	(5.2%)	.42
Life‐threatening arrhythmias	44	(5.7%)	55	(5.1%)	.58
Intubation	25	(3.2%)	36	(3.4%)	.90
Ejection fraction (%, median [IQR])	45 (40‐55)		50 (40‐55)		.013
Angiography performed	746	(96.8%)	1045	(97.0%)	.74
Vessel territory with disease^b^					
Left main disease	65	(8.7%)	88	(8.4%)	.83
LAD disease	461	(61.8%)	669	(64.0%)	.34
LCx disease	369	(49.5%)	493	(47.2%)	.34
RCA disease	416	(55.8%)	551	(52.7%)	.20
Vessel disease^b^					.46
Nonobstructive CAD	94	(12.6%)	119	(11.4%)	
One‐vessel disease	249	(33.4%)	386	(36.9%)	
Two‐vessel disease	191	(25.6%)	253	(24.2%)	
Three‐vessel disease	212	(28.4%)	287	(27.5%)	
Multivessel disease^b^	403	(61.8%)	540	(58.3%)	.16

Abbreviations: ACS, acute coronary syndrome; CABG, coronary artery bypass grafting; CAD, coronary artery disease; CVA, cerebrovascular accident; IQR, interquartile range; LAD, left anterior descending; LCx, left circumflex; MINOCA, myocardial infarction with nonobstructive CAD; NSTEMI, non‐ST‐segment elevation MI; PCI: percutaneous coronary intervention; STEMI, ST‐segment elevation MI; RCA, right coronary artery.

^a^ Percentage of missing data per characteristic/parameter: diabetes 3.2%; hypertension 3.1%; hypercholesterolemia 3.2%; smoking 3.1%; family history CAD 2.3%; chronic kidney disease 2.1%; peripheral arterial disease 2%; previous CVA 2.2%; previous MI 1.2%; previous CABG 1%; admission by interhospital transfer 0.2%; cardiogenic shock 0.1%; life‐threatening arrhythmias 0.1%; intubation 0.1%; ejection fraction 7.5%; radial access 0.4%.

^b^ Coronary artery disease status (ie, coronary anatomy) was not known in 57 patients (3.1%) who did not undergo coronary angiography.

### 3.1 ACS hospital admissions: comparison of COVID‐19 to control period

**FIGURE 1:**
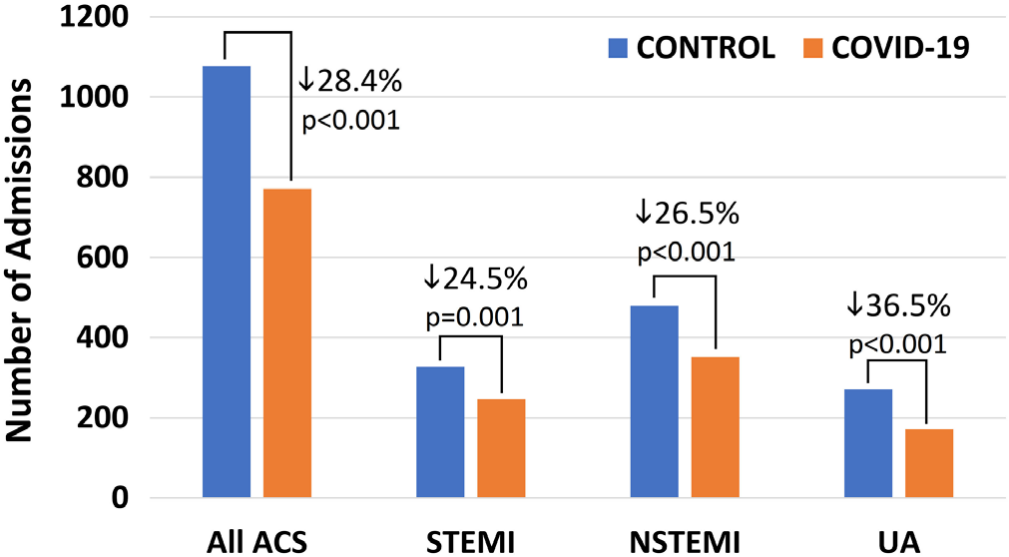
Number of acute coronary syndrome (ACS)‐related hospitalizations during the COVID‐19 outbreak (6‐weeks: 2 March 2020 to 12 April 2020 [orange bars]) are significantly reduced compared with the corresponding control period in 2019 (blue bars) overall and separately for ST‐segment elevation myocardial infarction (STEMI), non‐STEMI (NSTEMI) and unstable angina (UA). P values are derived from Poisson regression analysis

The mean rate of ACS admissions in the study period (18.4/day) was significantly lower compared to the control period (25.6 admissions/day; IRR: 0.72, 95% CI: 0.65‐0.79, *P* < .001). Table 2 reports the mean admission rates per day for all ACS types; IRRs in the study period were also significantly lower for each ACS type compared to the control period. These data translate to an overall 28.4% reduction of ACS hospitalizations in the study period (24.5% for STEMI, 26.5% for NSTEMI, and 36.5% for UA; Figure 1).

**TABLE 2:** Incidence rate of admissions for ACS in the COVID‐19 period compared with the control period

Admission diagnosis^a^	COVID‐19 (n = 771)	Control (n = 1077)	Incidence rate ratio (95% CI)	*P*‐value^b^
All ACS	18.4	25.6	0.72 (0.65–0.79)	<.001
STEMI	5.9	7.8	0.76 (0.64‐0.89)	.001
NSTEMI	8.4	11.4	0.73 (0.64‐0.84)	<.001
Unstable angina	4.1	6.5	0.63 (0.52‐0.77)	<.001
All ACS—week 1	24.6	25.1	0.98 (0.79‐1.21)	.83
All ACS—week 2	19.6	25.6	0.77 (0.61‐0.96)	.018
All ACS—week 3	17.1	23.9	0.72 (0.57‐0.91)	.006
All ACS—week 4	14.0	24.6	0.57 (0.44‐0.73)	<.001
All ACS—week 5	16.0	29.4	0.54 (0.43‐0.68)	<.001
All ACS—week 6	18.9	25.3	0.75 (0.60‐0.93)	.011

Abbreviations: ACS, acute coronary syndrome; CI, confidence interval; NSTEMI, non‐ST‐segment elevation MI; STEMI, ST‐segment elevation myocardial infarction.

^a^ Incidence rate is expressed as number of hospitalizations per day. Data for ACS are provided for the entire 6‐week period of observation and separately for each week. The incidence rate for each ACS type (STEMI, NSTEMI, unstable angina) for the entire 6‐week period is also provided.

^b^ To determine statistical significance for the comparison regarding (a) each one of the three ACS types and (b) each one of the 6 weeks of observation, the adjusted (Bonferroni correction for multiple comparisons) alpha levels of 0.017 (ie 0.05/3) and 0.008 (ie, 0.05/6) were used, respectively.

**FIGURE 2:**
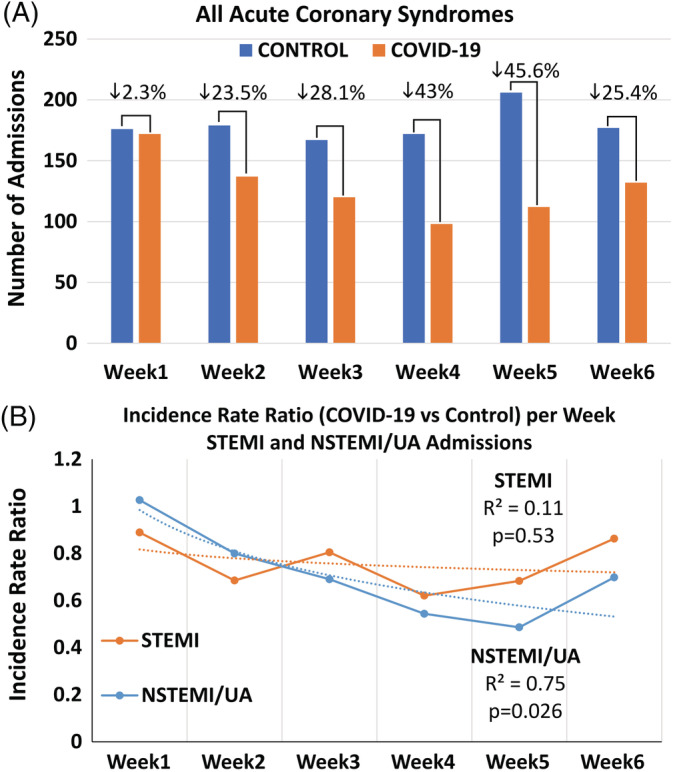
A, The overall number of admissions for acute coronary syndromes (ACS) on a week‐per‐week basis showed a gradual decline during the COVID‐19 period. B, The incidence rate ratio of admissions (ie, relative change in COVID‐19 compared with the control period) per week assessed separately for ST‐segment elevation myocardial infarction (STEMI) and non‐STEMI/unstable angina (UA) showed a temporal trend for NSTEMI/UA (*P* = .026) but not for STEMI (*P* = .53) which remained stable throughout the study period. Best‐fit curves (logarithmic) with the coefficients of determination (R^2^) are demonstrated

On a weekly basis, there was a gradual decline in all ACS admissions reaching a minimum of 14 and 16 admissions/day in the fourth and fifth week corresponding to a relative reduction of 43% and 46%, respectively, whereas the minimum rate observed in any week in the control period was 23.9 admissions/day (Table 2 and Figure 2A). After stratifying the incidence rate ratio (ie, the relative decrease in admissions) per week in the COVID‐19 compared to the control period according to STEMI and NSTEMI/UA, we found that the decrease in STEMI admissions was stable throughout the COVID‐19 period (temporal trend; *P* = .53), whereas there was a gradual decline in NSTEMI/UA admissions (temporal trend; *P* = .026) (Figure 2B).

### 3.2 ACS hospital admissions: the impact of the lockdown

**FIGURE 3:**
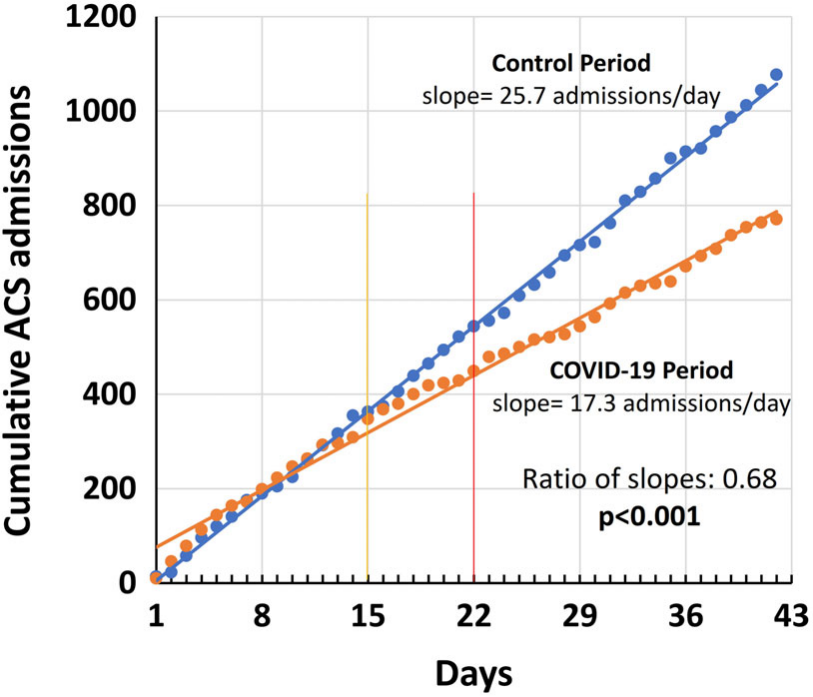
Cumulative number of acute coronary syndrome (ACS) hospitalizations plotted over the 42 days (6‐weeks) during the COVID‐19 outbreak (2 March 2020 to 12 April 2020 [orange]) and the corresponding period in 2019 (blue). The corresponding trend lines by linear regression are also shown. The numbers started to diverge substantially between March 16th (lockdown for over 80% of business type activities; yellow vertical line) and March 23rd (complete national lockdown with restriction of freedom of movement; red vertical line). There was an overall significant difference in the slopes of the regression lines between the COVID‐19 and control periods (17.3 vs 25.7 admissions/day, respectively; ratio of slopes: 0.68, 95% CI: 0.66‐0.70, *P* < .001)

The cumulative number of ACS hospitalizations plotted over the 6‐week observation period (42 days) for 2020 and 2019, and their respective trend lines (Figure 3), showed that the numbers started to diverge substantially between March 16th (lockdown for over 80% of business type activities) and March 23rd (national lockdown with restriction of freedom of movement). There was an overall significant difference in the slopes of the regression lines between the COVID‐19 and control periods (17.3 vs 25.7, respectively; ratio of slopes: 0.68, 95% CI: 0.66‐0.70, *P* < .001).

During the COVID‐19 study period, a reduction in the number of ACS admissions from 429 to 342 was observed following the complete national lockdown restricting the freedom of movement compared to the period before the lockdown (ie, the last 3 weeks compared with the first 3 weeks in the study). The mean rate of ACS admissions after the lockdown (16.3/day) was significantly lower compared to the period before the lockdown (20.4 admissions/day; IRR: 0.80, 95% CI: 0.69‐0.92, *P* = .002). The respective IRRs for the three ACS types (Table S1 in Appendix) indicated that the reduction after the lockdown was primarily due to a further decrease in NSTEMI admissions, while hospitalization rates for STEMI remained without significant change.

### 3.3 Older age and left ventricular systolic impairment

Patients admitted with ACS were less frequently above 65 years of age during the study period (44.2% of ACS admissions) compared to the control period (48.9% of ACS admissions; odds ratio: 0.83, 95% CI: 0.69‐0.99, *P* = .046). The respective results within each ACS type indicate that this difference was primarily due to the subgroup of patients presenting with unstable angina (Table S2in Appendix).

LV systolic impairment (defined as ejection fraction <40%) at ACS presentation (ie, a potential marker for delayed MI presentation) was registered more frequently during the COVID‐19 period compared to the control period (22.2 vs 15.5%; odds ratio: 1.56, 95% CI: 1.22‐1.99, *P* < .001). The separate results for each ACS type point out that this difference between the COVID‐19 and control period was particularly higher for STEMI patients and secondarily for NSTEMI patients, while there was no difference in UA patients (Table S2 in Appendix).

### 3.4 Treatment strategy for ACS

Overall, patients presenting with ACS underwent PCI less frequently during the COVID‐19 period compared to the control period (59.6 vs 64.4%; odds ratio: 0.82, 95% CI: 0.68‐0.99, *P* = .038). The proportion of STEMI patients undergoing primary PCI during the COVID‐19 period was lower compared to the control period (66.4 vs 74.0%; odds ratio: 0.69, 95% CI: 0.48‐0.99, *P* = .047) reflecting the numerically higher frequency of treatment with thrombolysis at the initial stage of STEMI presentation. Similarly, the proportion of patients with NSTEMI or UA undergoing PCI during the COVID‐19 period was also lower (48 vs 54.1% in the control period; odds ratio: 0.78, 95% CI: 0.62‐0.98, *P* = .031).

### 3.5 In‐hospital outcomes

In all ACS patients, there was a total of 54 in‐hospital deaths (3%) similarly distributed in the two periods (COVID‐19:3.3 vs control: 2.7%; odds ratio 1.20; 95% CI: 0.69‐2.06, *P* = .52). There was no difference in the rate of cardiac deaths between the two periods (2.6 % vs 2.4%; odds ratio 1.11; 95% CI: 0.61‐2.0, *P* = .74), while the rates of in‐hospital repeat MI (1% vs 0.4%; odds ratio 2.78; 95% CI: 0.83‐9.26, *P* = .083) and stent thrombosis (0.5% vs 0.2%; odds ratio 2.77; 95% CI: 0.51‐15.16, *P* = .25) were numerically higher during the COVID‐19 study period compared to the control one without reaching statistical significance.

## 4 DISCUSSION

The main findings of our study are summarized as follows: (a) there was a significant reduction in the rate of all ACS‐related admissions which was evident for all three ACS types (STEMI, NSTEMI and UA) in the COVID‐19 compared with the control period; (b) the decline of STEMI admissions was relatively stable throughout the COVID‐19 period, while the number of NSTEMI/UA admissions declined even more strikingly after the complete national lockdown; (c) the proportion of all ACS patients undergoing PCI as well as the proportion of STEMI patients treated with primary PCI was lower during the COVID‐19 period.

The overall reduction of ACS‐related admissions (28%) during the COVID‐19 outbreak in Greece is in line with contemporary reports about a 26% reduction in ACS hospitalizations in Northern Italy,^4^ a 39% reduction in STEMI/NSTEMI admissions in Austria,^2^ and a 38% reduction in STEMI activations in high‐volume cardiac catheterization laboratories in the USA.^5^ However, our observations come from a country with limited COVID‐19 community transmission; the incidence of COVID‐19 infections and related deaths in Greece at the end of our study period (13 April 2020) was only 20/100 000 and 0.92/100 000 inhabitants, respectively.^7^ As a result, the healthcare system did not face a huge overload in contrast to other European countries and the USA; hospitals were not deluged with COVID‐19 patients and the number of COVID‐19 cases treated in ICUs did not exceed 100 patients. The comparable decline in ACS hospitalizations in countries with both high and low penetrance of the SARS‐CoV‐2virus indicates that the overloaded health systems may not be the cause for this observation. The fact that the reduction of MI admissions was not linked to the lack of non‐COVID‐19 beds in Italy points to the same direction.^8^

Multiple factors may contribute to our observations regarding the decline in ACS admissions. First, this reduction may reflect a decreased contact of patients with the healthcare system. Fear among the public for potential exposure to SARS‐CoV‐2 and getting infected in a medical facility along with strict guidelines for self‐isolation may have inadvertently led many patients to avoid or delay seeking medical help. Supporting observations for this factor include the following. (a) There was a pronounced decline in the rate of admissions observed after the complete national lockdown only for ACS types with less severe clinical presentation/symptoms (ie, NSTEMI and UA); similar findings were observed during the national lockdown period in Northern Italy. (b) Older patients with an ACS (mainly UA) presented less frequently to the hospitals during the COVID‐19 period; older patients were considered from the start of the outbreak as one of the population groups at highest risk from COVID‐19 and were urged early by mass media and government authorities to avoid social contact and be self‐confined. (c) During the COVID‐19 outbreak, ACS patients presented more frequently with LV systolic impairment, which may serve as a surrogate marker for delayed MI presentation, and is supported by longer times from symptom onset to first medical contact observed for MI patients in other countries.^9^, ^10^, ^11^

An alternative cause for the decline in the rate of ACS hospitalizations is a true reduction in the incidence of ACS. This could be attributed to a lack of environmental triggers for MI due to the large‐scale measures of public and financial lockdown. Such measures decrease exposure to traffic, air pollution, and other respiratory infections (eg, influenza), all of which have been associated with acute MI.^12^, ^13^ Interestingly, after mid‐March 2020, the rates of influenza‐like illness were also reported to be lower compared with the same period in the previous year in Greece.^14^ A supporting finding of our study for a true reduction of ACS is that the observed reduction of STEMI remained stable during the COVID‐19 period and was not further influenced by the progressively stricter measures. The observed overall reduction of STEMI admissions is difficult to be explained simply by avoidance of seeking medical attention taking into account the severity of symptoms. In addition, according to epidemiological data during the pandemic in Greece,^6^ there was not a substantial increase in overall population mortality, which would have been expected if an excessive number of patients had suffered from ACS without seeking treatment.

Apart from the decline in ACS‐related admissions, we observed changes regarding the in‐hospital management of ACS with a relatively lower frequency of patients undergoing PCI for all ACS, including primary PCI for STEMI patients, which reflects a higher proportion of patients managed medically and a relatively higher use rate of thrombolysis in STEMI cases during the COVID‐19 pandemic. This is in line with reports from other countries about small increases in pharmacological thrombolysis and a decrease in the rate of PCI for NSTEMI patients.^3^, ^8^ During the COVID‐19 outbreak, multiple recommendations and protocols have been released advocating in some instances the use of thrombolysis in STEMI cases for suspected or confirmed COVID‐19 cases while keeping the PCI option only for selected very high‐risk ACS cases.^15^ The effect of these protocols on treatment decisions is not known.

The afore‐mentioned observations and speculations have important inferences for public health, government policies regarding the management of pandemics, and possibly social structure influencing triggering factors for ACS. Epidemiological data show that countries with low COVID‐19 incidence did not have a substantial change in mortality, whereas countries with high COVID‐19 incidence presented an excessive deviation in mortality from the expected level in most cases. In the latter, excessive deaths could be caused both by COVID‐19 and by collateral damage, such as in the case of ACS patients who may have avoided or did not receive proper treatment. In Northern Italy, an increase in mortality observed during the COVID‐19 outbreak could not be fully explained by COVID‐19 cases alone.^4^ During pandemics of infectious diseases, it would be essential that communication policies continue to encourage cardiovascular patients to seek routine medical care. Hospital services need to be designed so that they include (a) rapid ACS pathways for assessment and admission in separate areas from the ones used for patients suspected with infection and (b) point‐of‐care tests in the emergency department to rule out positive infection cases promptly, thereby facilitating transfer for urgent management without delays due to excessive safety precautions. These suggested changes may need to be designed and implemented soon in case of a second wave of the COVID‐19 pandemic. Finally, given the high likelihood of a truly reduced ACS incidence during this unexpected experiment of social restrictions and quarantining, further research is needed on the pathophysiology of ACS and any modifiable environmental factors, and potentially how social life could be restructured so that triggering factors and the associated ACS incidence could be reduced.

### 4.1 Limitations

The data presented are observational and no definitive conclusions can be drawn about causality. This is a retrospective study with all shortcomings associated with such an approach including the lack of additional variables of interest, which were not readily available. However, our study gathered comparative data over a 6‐week period from the huge majority of hospitals with primary PCI services in the country, thereby providing representative information on ACS‐related hospitalizations.

## 5 CONCLUSIONS

We observed a decline in ACS‐related hospitalizations during the COVID‐19 outbreak in a country with low penetrance of SARS‐CoV‐2 virus, strict measures of social restrictions and no substantial increase in overall population mortality. Our findings provide indirect evidence that medical care avoidance behavior among ACS patients is an important factor for these observations, while a true reduction of the ACS incidence due to self‐isolation/quarantining, and thus, a lack of environmental triggers for ACS, cannot be excluded. Further research is needed to clarify these factors.

## CONFLICT OF INTEREST

The authors declare no potential conflict of interest.

## Supporting information


**Appendix** **S1.** Supporting Information.

## References

[1] Huang C , Wang Y , Li X , et al. Clinical features of patients infected with 2019 novel coronavirus in Wuhan, China. Lancet. 2020;395:497‐506. 31986264 10.1016/S0140-6736(20)30183-5 PMC7159299

[2] Metzler B , Siostrzonek P , Binder RK , Bauer A , Reinstadler SJ . Decline of acute coronary syndrome admissions in Austria since the outbreak of COVID‐19: the pandemic response causes cardiac collateral damage. Eur Heart J. 2020;41:1852‐1853. 32297932 10.1093/eurheartj/ehaa314 PMC7184486

[3] Rodriguez‐Leor O , Cid‐Alvarez B , Ojeda S , et al. Impact of the COVID‐19 pandemic on interventional cardiology activity in Spain. REC Interv Cardiol. 2020;2:82‐89.

[4] De Filippo O , D'Ascenzo F , Angelini F , et al. Reduced rate of hospital admissions for ACS during Covid‐19 outbreak in Northern Italy. N Engl J Med. 2020;383(1):88–89. 32343497 10.1056/NEJMc2009166 PMC7224608

[5] Garcia S , Albaghdadi MS , Meraj PM , et al. Reduction in ST‐segment elevation cardiac catheterization laboratory activations in the United States during COVID‐19 pandemic. J Am Coll Cardiol. 2020;75:2871‐2872. 32283124 10.1016/j.jacc.2020.04.011 PMC7151384

[6] EuroMOMO hub . European mortality monitoring activity. https://euromomo.eu/graphs-and-maps. 2020.

[7] National Public Health Organization . Daily report on epidemiological surveillance for new coronavirus disease (COVID‐19). https://eody.gov.gr/wp-content/uploads/2020/04/covid-gr-daily-report-20200413-1.pdf Accessed April 13, 2020 (In Greek).

[8] De Rosa S , Spaccarotella C , Basso C , et al. Reduction of hospitalizations for myocardial infarction in Italy in the COVID‐19 era. Eur Heart J. 2020;41:2083‐2088. 32412631 10.1093/eurheartj/ehaa409 PMC7239145

[9] Tam CF , Cheung KS , Lam S , et al. Impact of coronavirus disease 2019 (COVID‐19) outbreak on ST‐segment‐elevation myocardial infarction care in Hong Kong, China. Circ Cardiovasc Qual Outcomes. 2020;13:e006631. 32182131 10.1161/CIRCOUTCOMES.120.006631 PMC7147280

[10] Abdelaziz HK , Abdelrahman A , Nabi A , et al. Impact of COVID‐19 pandemic on patients with ST‐segment elevation myocardial infarction: insights from a British cardiac center. Am Heart J. 2020;226:45‐48. 32497914 10.1016/j.ahj.2020.04.022 PMC7211651

[11] Toner L , Koshy AN , Hamilton GW , Clark D , Farouque O , Yudi MB . Acute coronary syndromes undergoing percutaneous coronary intervention in the COVID‐19 era: comparable case volumes but delayed symptom onset to hospital presentation. Eur Heart J Qual Care Clin Outcomes. 2020;7:qcaa038. 10.1093/ehjqcco/qcaa038. Online ahead of print. PMC7239230 32379888

[12] Nawrot TS , Perez L , Kunzli N , et al. Public health importance of triggers of myocardial infarction: a comparative risk assessment. Lancet. 2011;377:732‐740. 21353301 10.1016/S0140-6736(10)62296-9

[13] Kwong JC , Schwartz KL , Campitelli MA , et al. Acute myocardial infarction after laboratory‐confirmed influenza infection. N Engl J Med. 2018;378:345‐353. 29365305 10.1056/NEJMoa1702090

[14] Flu News Europe . Joint ECDC‐WHO/Europe weekly influenza update. https://flunewseurope.org/CountryData?country=EL. 2020.

[15] Zeng J , Huang J , Pan L . How to balance acute myocardial infarction and COVID‐19: the protocols from Sichuan provincial people's hospital. Intensive Care Med. 2020;46:1111‐1113. 32162032 10.1007/s00134-020-05993-9 PMC7079823

