# Supplementary material for: “Missing” acute coronary syndrome hospitalizations during the COVID‐19 era in Greece: Medical care avoidance combined with a true reduction in incidence?
Source: Clin Cardiol. 2020 Jul 21;43(10):1142–9. doi: 10.1002/clc.23424 (PMC7404667; doi:10.1002/clc.23424)
Supplement: Supplementary file 1 — Appendix S1. Supporting Information. [file CLC-43-1142-s001.docx]

**Online Supplement**

**“Missing” acute coronary syndrome hospitalizations during the COVID-19 era in Greece: Medical care avoidance combined with a true reduction in incidence?**

**SUPPLEMENTAL METHODS**

**Data and Definitions**

Acute coronary syndrome diagnoses, demographics, clinical and angiographic characteristics, information on management and PCI characteristics, and in-hospital outcomes were retrospectively retrieved from patients’ hospital records, discharge letters and records of catheterization laboratories. Classification in STEMI, NSTEMI and UA was done in compliance with the current guidelines.^1, 2^ After gathering data from all hospitals in a central electronic database, data were finally checked for missing or contradictory entries, and necessary corrections were applied in communication with each hospital.

Critical clinical condition at ACS presentation was assessed by the presence of cardiogenic shock, life-threatening arrhythmias and intubation. A vessel was considered diseased when a luminal narrowing of ≥50% diameter stenosis in a major epicardial artery or one of its major branches was present. Multivessel disease was defined when more than one major coronary artery systems were diseased. Left main disease was considered to be equivalent to two-vessel disease. MINOCA was defined as MI (STEMI or NSTEMI) with nonobstructive coronary artery disease (i.e. lesions with <50% diameter stenosis in any vessel).

**SUPPLEMENTAL RESULTS**

A total of 19 public hospitals with PCI capability participated in the study representing more than 80% of the PCI volume performed in all Greek public hospitals. Of these, seven (37%) were COVID-19 dedicated hospitals.

A total of 1848 ACS patients were included in the study (771 during the study period and 1077 during the control period). During the study period there were only 31 ACS patients suspected for COVID-19 at presentation and none confirmed with the disease. **Table 1** in the main manuscript shows the demographics, clinical characteristics, and angiographic data of the enrolled patients. Most patients were male (77.4%) with a median age of 65 (IQR: 56-74) years old. There were no outstanding differences in the registered baseline characteristics between the two periods except for the lower ejection fraction of patients presenting with ACS during the COVID-19 period.

The largest percentage of patients were admitted with NSTEMI (n=831, 45%) followed by patients with STEMI (n=574, 31.1%); the distribution of ACS type was similar in the two periods. The proportion of patients transferred by referral hospitals (i.e. admission by interhospital transfer) was similar in the COVID-19 (34%) and control (32.8%) periods. Critical clinical condition as reflected by cardiogenic shock, life-threatening arrhythmias or need for intubation during ACS presentation was infrequent in both the COVID-19 and control periods.

Coronary angiography rates (96.8 vs. 97%) as well as the rates of MI with non-obstructive coronary artery disease (MINOCA; 7.1 vs 7.3%) were similar between the study and control period, respectively. Angiographic findings were comparable between the study and control period.

**SUPPLEMENTAL REFERENCES**

1. Roffi M, Patrono C, Collet JP, et al. 2015 ESC Guidelines for the management of acute coronary syndromes in patients presenting without persistent ST-segment elevation: Task Force for the Management of Acute Coronary Syndromes in Patients Presenting without Persistent ST-Segment Elevation of the European Society of Cardiology (ESC). *Eur Heart J*. 2016; 37:267-315.

2. Ibanez B, James S, Agewall S, et al. 2017 ESC Guidelines for the management of acute myocardial infarction in patients presenting with ST-segment elevation: The Task Force for the management of acute myocardial infarction in patients presenting with ST-segment elevation of the European Society of Cardiology (ESC). *Eur Heart J*. 2018; 39:119-177.

**Online Table 1S. Incidence rate of admissions for ACS during the COVID-19 period before and after complete national lockdown***

| **Admission Diagnosis**† | **After Lockdown (n=342)** | **Before Lockdown (n=429)** | **Incidence Rate Ratio (95% CI)** | **P value**‡ |
| --- | --- | --- | --- | --- |
| All ACS | 16.3 | 20.4 | 0.80 (0.69-0.92) | 0.002 |
| STEMI | 5.8 | 6.0 | 0.96 (0.75-1.23) | 0.750 |
| NSTEMI | 7.0 | 9.8 | 0.71 (0.57-0.88) | 0.001 |
| UA | 3.6 | 4.6 | 0.77 (0.57-1.05) | 0.094 |

*Complete national lockdown with restriction of the freedom of movement was implemented on the 23^rd^ of March 2020. Accordingly, “Before Lockdown” represents the first 3 weeks (March 2 to 22, 2020) and “After Lockdown” represents the last 3 weeks (March 23 to April 12, 2020) during the COVID-19 study period

†Incidence rate is expressed as number of hospitalizations per day

‡To determine statistical significance for the comparison regarding each one of the three ACS types, the adjusted (Bonferroni correction for multiple comparisons) alpha level of 0.017 (i.e. 0.05/3) was used

ACS, acute coronary syndrome; CI, confidence interval; NSTEMI, non–ST-segment elevation myocardial infarction; STEMI, ST-segment elevation myocardial infarction; UA, unstable angina

**Online Table 2S. Older age and left ventricular systolic dysfunction in patients admitted with ACS in the COVID-19 compared with the control period**

| **Admission Diagnosis** | **Subgroup** | **Frequencies COVID-19 vs Control** | **Odds Ratio** | **95% CI** | **P value** |
| --- | --- | --- | --- | --- | --- |
| **All ACS** | Age>65 years | 44.2 vs 48.9% | 0.83 | 0.69-0.99 | 0.046 |
|  | EF<40% | 22.2% vs 15.5% | 1.56 | 1.22-1.99 | <0.001 |
| **STEMI** | Age>65 years | 37.2 vs 40.7% | 0.87 | 0.62-1.22 | 0.41 |
|  | EF<40% | 31 vs 19.7% | 1.83 | 1.24-2.70 | 0.002 |
| **NSTEMI** | Age>65 years | 48.6 vs 50.7% | 0.92 | 0.70-1.21 | 0.54 |
|  | EF<40% | 21.8 vs 16.1% | 1.46 | 1.01-2.09 | 0.041 |
| **UA** | Age>65 years | 45.3 vs 55.7% | 0.66 | 0.45-0.97 | 0.033 |
|  | EF<40% | 10.2 vs 8.1% | 1.29 | 0.64-2.62 | 0.47 |

ACS, acute coronary syndrome; CI, confidence interval; EF, ejection fraction; NSTEMI, non–ST-segment elevation myocardial infarction; STEMI, ST-segment elevation myocardial infarction; UA, unstable angina
